# Genomic analyses of an *Escherichia coli* and *Klebsiella pneumoniae* urinary tract co‐infection using long‐read nanopore sequencing

**DOI:** 10.1002/mbo3.1396

**Published:** 2024-01-17

**Authors:** Stephen Mark Edward Fordham, Magdalena Barrow, Anna Mantzouratou, Elizabeth Sheridan

**Affiliations:** ^1^ Department of Life & Environmental Sciences, Talbot Campus Fern Barrow Bournemouth University Poole UK; ^2^ Department of Medical Microbiology, Poole Hospital University Hospitals Dorset NHS Foundation Trust Poole England

**Keywords:** IS*26*, plasmid, resistance, urinary tract infection

## Abstract

*Escherichia coli* and *Klebsiella pneumoniae* isolates presenting with the same antimicrobial susceptibility profile were recovered from the same catheter sample of urine (CSU). Both strains were recovered from a patient with a long‐standing indwelling urinary catheter. Each isolate had its DNA extracted following culture. Nanopore long‐read sequencing was used to build the plasmids and chromosomes from each strain to closure to discern the potential horizontal propagation of resistance‐encoding plasmids and the relationship between resistance genes and insertion sequences. Plasmids derived from resistance strains in the urinary microbiota remain poorly characterized. The same 11 antimicrobial resistance (AMR) genes were found in plasmids from each strain. The 185,239‐bp FIB(K) pKBM1, from the *K. pneumoniae* strain, additionally encoded the five AMR genes: *sul2, strA, strB, bla*_TEM‐1B_, and *bla*_CTX‐M‐15_. A multimeric array of AMR genes and IS*26* insertion sequences were found in the plasmids from both isolates. Both plasmids from each isolate were similar. Horizontal transfer of plasmids, followed by subsequent plasmid rearrangement, is likely to have occurred during infection. Furthermore, the resistance region in the plasmids shared similarity against the internationally prevalent plasmid, pKPN3‐307_typeA, commonly identified in *K. pneumoniae* ST307. Biofilm formation in catheterized patients may allow close cell contact between strains. Horizontal propagation of resistance genes may occur, leading to polymicrobial infections.

## Graphical abstract

Two bacterial strains were recovered from the same catheter specimen of urine sample. The *Escherichia* coli strain encoded 11 resistance genes on a plasmid, including the ESBL gene, *bla*_CTX‐M‐15._ The *Klebsiella pneumoniae* strain encoded an additional 5 resistance genes on a plasmid, including 2 copies of *bla*_CTX‐M‐15._ Resistance genes were flanked between IS*26* in a multimeric array. Both plasmids were similar, suggesting horizontal transfer occurred during the course of infection.

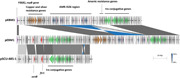

## 1 INTRODUCTION

Urinary tract infections (UTIs) associated with the use of an indwelling catheter represent one of the most common infections encountered in hospital settings (Nicolle, 2014). The prolonged duration of catheterization has been associated with biofilm formation, which can form a barrier resistant to antibiotic penetration (Ramadan et al., 2021). These indwelling catheters and their associated biofilms represent an important reservoir for Gram‐negative pathogens including both *Escherichia coli* and *Klebsiella pneumoniae*. Recently, 4.1% of UTI cases represented co‐infections of *E. coli* and *K. pneumoniae* strains (Ndzime et al., 2021).

The horizontal spread of extended‐spectrum beta‐lactamase (ESBL) encoding plasmids represents an important mechanism mediating resistance to cephalosporins in both *K. pneumoniae* and *E. coli* (Agyekum et al., 2016; Evans et al., 2020; Pedersen et al., 2020). In vitro assays have confirmed the conjugation of urinary plasmids sourced from *E. coli* isolates with separate F‐ and P‐type conjugative transfer genes into the recipient *E. coli* K‐12 strain MG1655 (Hernandez et al., 2022). Moreover, conjugative genes have also been found in plasmid assemblies derived from urinary *E. coli* and *K. pnuemoniae* isolates, suggesting these plasmids may be mobile (Hernandez et al., 2021). Horizontal gene transfer (HGT) via plasmid‐mediated conjugation of antimicrobial resistance genes (AMR) can occur at a higher rate in biofilms, relative to planktonic cultures (Savage et al., 2013). Despite this, complete plasmids from the urinary microbiota and their potential for resistance dissemination remain poorly understood.

In addition to the horizontal propagation of plasmids, the insertion sequence (IS) IS*26* contributes to both the recruitment and dissemination of AMR genes (He et al., 2015). IS*26* is prevalent in antibiotic‐resistant Gram‐negative pathogens including *E. coli* and *K. pneumoniae* (Ge et al., 2022; Luo et al., 2022; Weber et al., 2019). AMR genes are frequently found within IS*26*‐bounded structures (Zhao et al., 2021). This configuration, which must include directly oriented IS*26* copies, which each encode TnpA26 transposases are called pseudo‐compound transposons (PCTs). Each IS*26* is bounded by perfect 14‐bp terminal inverted repeats (TIRs), including inverted repeat left (IRL) and inverted repeat right (IRR) sequences.

Separate *E. coli* and *K. pneumoniae* strains presenting with the same antimicrobial susceptibility profile were recovered at the same time from the same catheter sample of urine (CSU), from a patient with a long‐standing indwelling urinary catheter from a UK district general hospital. We undertook a long‐read sequencing approach to determine whether the same or portions of a resistance encoding plasmid were shared between the two strains to discern potential HGT. Using the genomic context provided by long‐read assembly, we further investigated the relationship between resistance genes and IS*26*.

## 2 METHODS

### 2.1 Culturing and antibiotic susceptibility testing (AST)

*E. coli* and *K. pneumoniae* isolates were recovered from a patient from a UK district general hospital. The patient presented with a UTI associated with a long‐standing indwelling urinary catheter. Before the sample was cultured, the patient had received courses of antibiotics including an intravenous second‐generation cephalosporin and an intravenous ureidopenicillin/beta‐lactamase inhibitor combination.

*E. coli*/*K. pneumoniae* samples obtained from the same CSU sample were cultured onto cysteine‐, lactose‐, and electrolyte‐deficient (CLED) agar (Sigma‐Aldrich) to support the growth of urinary pathogens. Agar plates were supplemented with 8 µg/mL cefotaxime (Sigma‐Aldrich) to encourage the maintenance of any potential ESBL‐encoding plasmid(s). The strains were grown overnight at 37°C.

A single colony from each strain was added onto a Matrix‐Assisted Laser Desorption/Ionization (MALDI) target plate. A total of 1 µL of α‐Cyano‐4‐hydroxycinnamic acid (CHCA) matrix solution prepared with 50% acetonitrile and 2.5% trifluoroacetic acid in pure water was applied onto the smear. A MALDI Time‐of‐Flight Mass Spectrometry (MALDI‐TOF MS) (Bruker Daltonics) assignment score ≥2.0 was used to confirm species identification.

Separate *E. coli* and *K. pneumoniae* colonies were inoculated into 10 mL of lysogeny broth (LB) containing 8 µg/mL cefotaxime (Sigma‐Aldrich) in a 50 mL centrifuge tube to permit aerobic respiration. The culture grew at 37°C for 16 h. Total bacterial DNA, including chromosomal and plasmid DNA, was extracted using the Wizard® HMW DNA extraction kit from Promega (Southampton) following instructions provided by the manufacturer.

Antimicrobial susceptibility testing (AST) was performed using the Kirby–Bauer disc diffusion assay. Multidrug resistance (MDR) was defined by either nonsusceptibility or resistance to at least one agent from three or more antibiotic classes (Magiorakos et al., 2012). *E. coli* ATCC 25922 was used as the quality control strain.

The ceftriaxone minimum inhibitory concentration (MIC) was determined for each isolate to decipher ESBL production. Bacteria from beads stored in glycerol were inoculated onto Mueller‐Hinton agar plates and grown overnight at 37°C. Two to three morphologically similar bacterial colonies were lightly touched using the Prompt inoculation system wand (BioMérieux) and suspended thoroughly into a blank saline solution. The turbidity of the inoculum in the saline solution was standardized to 0.5 McFarland units. A sterile swab was inserted into the bacterial suspension and streaked onto a Mueller–Hinton agar plate to create a lawn. The BioMérieux ETEST AST reagent strip, ceftriaxone (TXL 0.002‐32) was applied to the plates using aseptic techniques and incubated for 24 h at 37°C. The MIC was recorded where the zone of inhibition intercepts the AST strip. Interpretation of sensitivity zone diameter and MIC breakpoints was determined using the 2023 European Committee on Antimicrobial Susceptibility Testing (EUCAST) Version 13.0 document guidelines (EUCAST, 2023).

### 2.2 Genome assembly

Downstream library preparation was performed using the PCR‐free Rapid sequencing gDNA barcoding kit, SQK‐RBK004 (Oxford Nanopore Technologies). Long‐read sequencing was performed using the MinION Mk1B sequencer (ONT) using default settings. The prepared library was loaded onto a R9.4.1 flowcell (ONT). Raw Fast5 was base‐called using Guppy version 6.3.8 using the high‐accuracy, r9.4.1_450bps_hac model. FASTQ files were binned into their respective barcode folder. De novo genome assembly was performed using Flye v2.9 (Kolmogorov et al., 2019), following light read filtering which discarded reads <1000‐bp. Graph assembly files for each final genome assembly were visualized using Bandage v0.8.1 to confirm the circularity of chromosomes and plasmids. Flye draft genome assemblies were polished consecutively using two rounds of post‐assembly polishing using Medaka v1.6.0 (https://github.com/nanoporetech/medaka).

### 2.3 Genome annotation and typing

Bacterial whole genome annotation was performed using Prokka v1.14.6 (Seemann, 2014). Plasmid replicon typing was performed using PlasmidFinder, employing a minimum identity and coverage of 98% (Carattoli & Hasman, 2020). The plasmid typing database from PubMLST (https://pubmlst.org/bigsdb?db=pubmlst_plasmid_seqdef) was used for further sub‐typing of FII plasmid replicons. In silico antimicrobial resistance prediction was performed using ResFinder 4.0 with a minimum nucleotide identity and length of 99% (Zankari et al., 2012). MobileElementFinder v.1.0.2 available from the Center for Genomic Epidemiology (https://www.genomicepidemiology.org/) was used to identify mobile genetic elements (MGEs) and their relationship with AMR genes. Sequence type was assigned using the Multi Locus Sequence Typing Tool (MLST v2.0) available from the Center for Genomic Epidemiology (https://www.genomicepidemiology.org/).

## 3 RESULTS

### 3.1 Strain summary

Two strains were recovered from a CSU sample from a patient presenting with a long‐standing (>3 months) indwelling urinary catheter from a UK district general hospital. An overview of the strains and their resistance gene profile is shown in Table 1.

**Table 1:** Strain summary.

	KC1	pKBM1	EC_01	pEBM1
Contig	Chromosome	Plasmid	Chromosome	Plasmid
Plasmid replicon type	‐	FIB(K)	‐	FIB(K) and FII‐29
Length (bp)	5,230,307 bp	185,239 bp	5,054,179 bp	247,127 bp
AMR genes	**3**: *bla*_SHV‐126_, *oqxA, oqxB*	**16**: *aac(3)‐IIe, aac(6’)‐Ib‐cr, strA* (2 copies), *strB* (2 copies), *bla*_CTX‐M‐15_ (2 copies), *bla*_OXA‐1_, *bla*_TEM‐1B_ (2 copies), *dfrA14, qnrB1, sul2* (2 copies), and *tet(A)*	‐	**11**: *aac(3’)‐IIe, aac(6’)‐Ib‐cr, strA, strB, bla*_CTX‐M‐15_, *bla*_OXA‐1_, *bla*_TEM‐1B_, *dfrA14, qnrB1, sul2*, and *tet(A)*
GenBank Accession	CP119564.1	CP119565.2	CP120234.1	CP120235.2

The *K. pneumoniae* strain was typed as ST1564‐1LV, with a K locus type (KL) 126 and the O locus type (OL) OL101. The *K. pneumoniae* strain, KC1, harbored a single plasmid, pKBM1 of 185,239‐bp assembled with an average read depth of 148. pKBM1 was typed as a FIB(K) plasmid carrying the following 16 AMR genes: *aac(3)‐IIe, aac(6*′*)‐Ib‐cr, strA* (two copies), *strB* (two copies), *bla*_CTX‐M‐15_ (two copies), *bla*_OXA‐1_, *bla*_TEM‐1B_ (two copies), *dfrA14, qnrB1, sul2* (two copies), and *tet(A)*, respectively. The KC1 genotype correlated with the resistant phenotype. KC1 was resistant to the following 10 antibiotics: gentamicin, tobramycin (*strA, strB, aac(6*′)*‐Ib‐cr*), ciprofloxacin (*qnrB1, aac(6*′*)‐Ib‐cr*), amoxicillin‐clavulanic acid, ampicillin, ceftazidime, cefpodoxime, cefuroxime, piperacillin‐tazobactam (*bla*_OXA‐1_, *bla*_TEM‐1B_, *bla*_CTX‐M‐15_), and trimethoprim‐sulfamethoxazole (*sul2, dfrA14*), respectively.

The *E. coli* strain, EC_01 was typed as ST127, O6:H31. EC_01 encoded a single plasmid of 241,127‐bp, pEBM1 assembled with a read depth of 281. pEBM1 carries the following 11 AMR genes: *aac(3)‐IIe, aac(6*′*)‐Ib‐cr, strA, strB, bla*_CTX‐M‐15_, *bla*_OXA‐1_, *bla*_TEM‐1B_, *dfrA14, qnrB1, sul2*, and *tet(A)*, respectively. pEBM1 contains two replicons. Replicon sequence typing identified FIB(K), and FII replicons, respectively. The FII replicon was further sub‐typed to FII‐29 using PubMLST. EC_01 also had a genotype that correlated with the strain's phenotypic resistance. EC_01 was resistant to the following 10 antibiotics: gentamicin, tobramycin, (*strA, strB, aac(6*′*)‐Ib‐cr*), ciprofloxacin (*qnrB1, aac(6*′*)‐Ib‐cr*), amoxicillin‐clavulanic acid, ampicillin, ceftazidime, cefpodoxime, cefuroxime, piperacillin‐tazobactam (*bla*_OXA‐1_, *bla*_TEM‐1B_, *bla*_CTX‐M‐15_), trimethoprim‐sulfamethoxazole (*sul2, dfrA14*). The reference control strain, *E. coli* ATCC 25922 was susceptible to all the antibiotics tested. Both KC1 and EC_01 had a MIC to ceftriaxone ≥32 mg/L, surpassing the 2 mg/L resistance threshold classification for Enterobacterales (EUCAST, 2023).

### 3.2 Plasmid backbone

**Figure 1:**
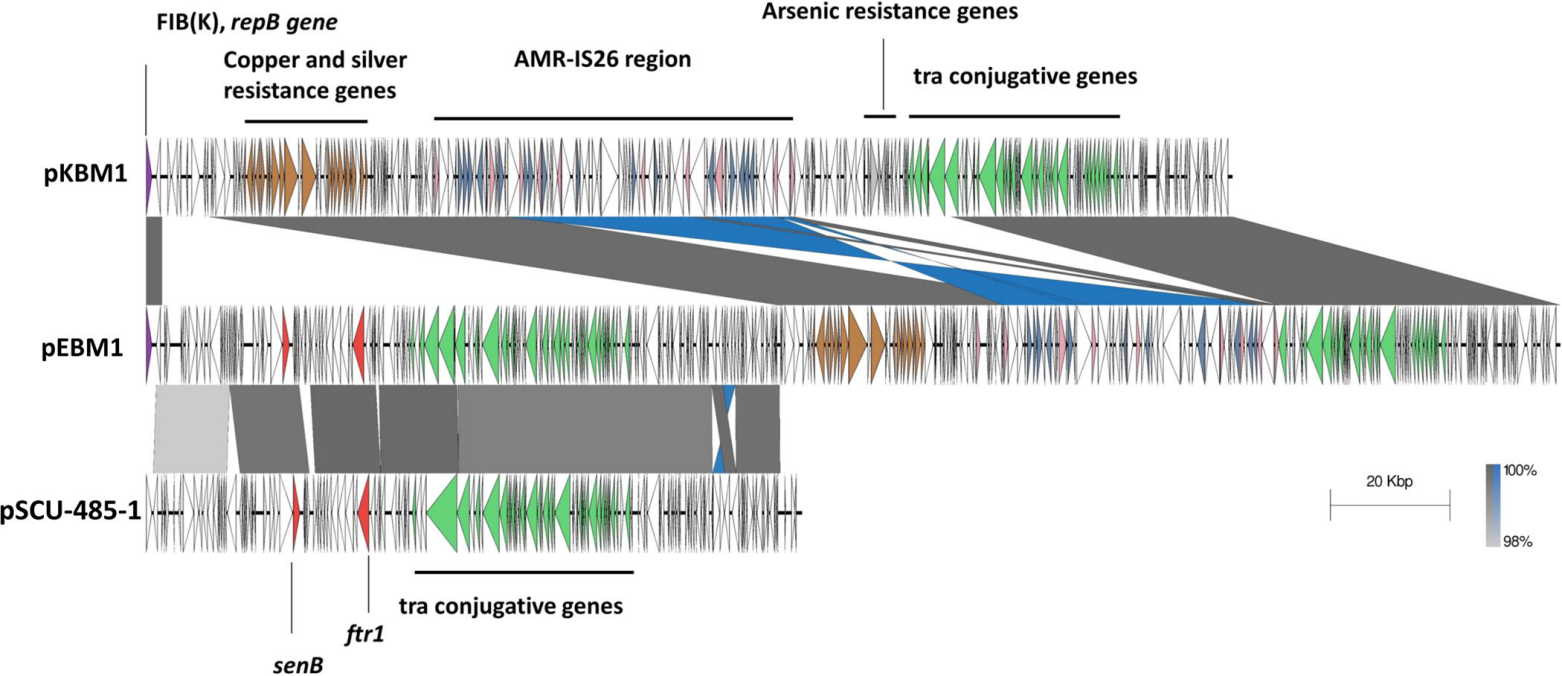
pEBM1 (middle) comparison against both pKBM1 (top) and pSCU‐485‐1 (bottom, accession: CP053246.1). For plasmid comparison, both pEBM1 and pKBM1 were aligned at the same shared start gene, *repB* (shaded purple), encoding the RepFIB replication protein B with the same orientation. pEBM1 comprises regions belonging to both pKBM1 and pSCU‐485‐1. The MDR region is inverted in pKBM1 relative to pEBM1. Gene regions are shown as annotated arrows. Grey gene synteny blocks depict regions with >99% identity with the same orientation, blue blocks represent inverted regions with >99% identity. Green genes represent *tra* conjugative transfer genes, blue genes indicate AMR genes and pink genes indicate IS*26*‐containing *tnpA26* transposase genes. Red genes include both the enterotoxin *senB* and the iron permease gene *ftr1*, derived from the pSCU‐485‐1‐like plasmid. Grey genes in pKBM1 correspond to arsenic resistance genes which are absent in pEBM1. Genome comparisons were performed using Easyfig v.2.2.5 (Sullivan et al., 2011).

pEBM1 is a cointegrate formed by the fusion of two plasmids. These include regions belonging to both the FII‐29 pSCU‐485‐1‐like plasmid (accession: CP053246.1) and the FIB(K) pKBM1 (Figure 1). pEBM1 contains an insertion of 105,139‐bp, relative to pKBM1. The insertion is similar to plasmid pSCU‐485‐1 (accession: CP053246.1). pSCU‐485‐1, length 111,831‐bp, has 48% query coverage and 99.52% identity against the 241,127‐bp pEBM1 (Figure 1). Both plasmids from the 2 isolated strains also shared an identical 1011‐bp *repB* gene encoding a 336 amino acid RepFIB replication protein B.

Plasmid replicon typing identified two replicons carried by pEBM1. These included a shared FII‐29 replicon subtype between pEBM1 and pSCU‐485‐1, alongside an identical FIB(K) replicon shared with pKBM1 (Figure 1). Both pEBM1 and pSCU‐485‐1 share a complete *tra* transfer operon, *traMJYALEKBPGVRCWUNFQHGSTDI‐traX* downstream of an origin of transfer (*oriT*) site. In addition, both plasmids harbor the virulence‐associated gene, *senB*, encoding an enterotoxin, and the iron permease gene, *ftr1*.

The 185,239‐bp FIB(K) pKBM1 shares 59% query coverage and 99.90% identity against pEBM1. Both pEBM1 and pKBM1 share heavy metal resistance genes (HMRGs) in a 52.8‐kb shared region. These include genes encoding the copper cation efflux system *cusSRCFBA*, copper resistance genes (*copA, copB, copC, and copD*), and the silver resistance gene, *silE* (Figure 1). In contrast to the HMRG region, the 46,188‐bp pEBM1 MDR region except for the final bracketing *tnpA26* gene upstream of the Tn2 fragment is inverted in pKBM1. While the MDR region in pKBM1 is proceeded by a 26,683‐bp region encoding the arsenic resistance genes, *arsB, arsC*, and *arsH*, followed by the conjugative transfer genes *finO, traX, traI*, and *traD*, this region is absent in pEBM1, leading to an interrupted *tra* transfer region. The fact that *E. coli* and *K. pneumoniae* hosts of pEBM1 and pKBM1 were isolated from the same patient might suggest that HGT occurred before cointegrate formation.

### 3.3 Additional IS*26* PCT formation in pKBM1

**Figure 2:**
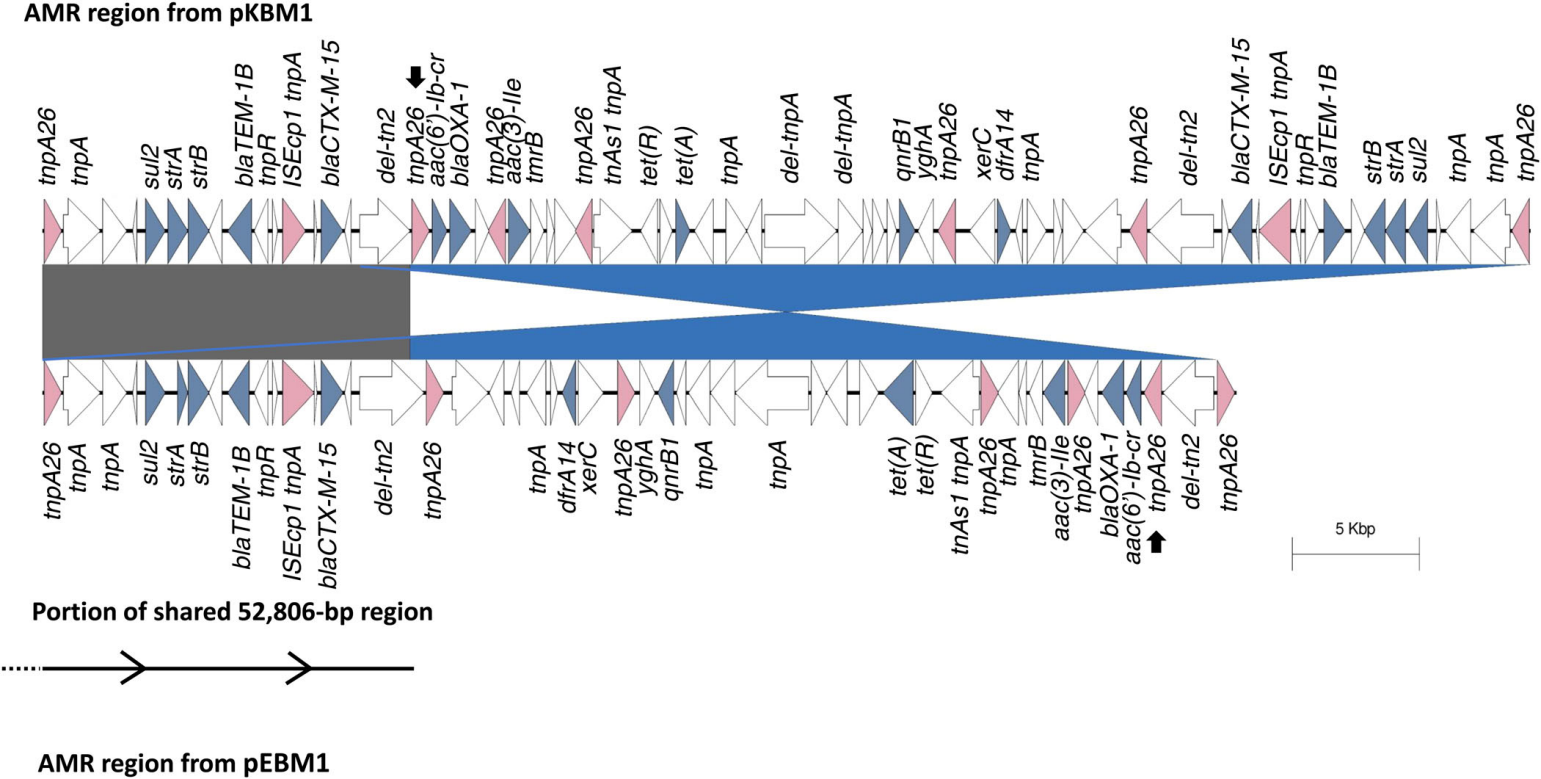
Antimicrobial resistance (AMR) resistance region comparison between pKBM1 and pEBM1. The entire resistance gene region in pEBM1 (bottom) except for the rightmost IS*26* encoding the transposase TnpA26 is inverted relative to pKBM1 (top). Upstream of this inversion in pKBM1, a 52,806‐bp fragment from pEBM1 is present. Within this 52.8‐kb region, an IS*26* and the following five AMR genes are found: *sul2, strA, strB, bla*_TEM‐1B_, and *bla*_CTX‐M‐15_. This is indicated by the grey gene synteny block. The IS*26* encoding *tnpA26* and the five AMR genes are located upstream of a second directly orientated IS*26* copy, indicated by a black arrow. The corresponding IS*26*‐encoded *tnpA26* on the inverted region in pEBM1 is indicated by a black arrow. The presence of the 52,806‐bp region encoding the five AMR genes combined with the IS*26* present in the inversion region forms a complete 15,301‐bp IS*26* PCT harboring the *sul2, strA, strB, bla*_TEM‐1B_, and *bla*_CTX‐M‐15_. Regions with > 99% identity are shown. Inversion is shown in blue. Genome comparisons were performed using Easyfig v.2.2.5 (Sullivan et al., 2011).

The complete resistance regions between pKBM1 and pEBM1 were compared against one another. The resistance regions were associated with IS*26* insertion sequences. The region encoding resistance genes on pKMB1 was 58,609‐bp, 11,597‐bp longer than the 47,012‐bp nucleotide stretch encoding resistance genes on pEBM1 (Figure 2). pKBM1 additionally encodes the five AMR genes: *sul2, strA, strB, bla*_TEM‐1B_, and *bla*_CTX‐M‐15_, respectively alongside an IS*26*. The region encoding resistance genes and their associated IS*26* copies are inverted in pKBM1, relative to pEBM1. Upstream of this inversion in pKBM1, a 52,806‐bp fragment from pEBM1 is present. This fragment forms an additional 15,301‐bp IS*26* PCT encoding *bla*_CTX‐M‐15_ (Figure 2).

### 3.4 Multimeric array of IS*26* and AMR genes in pKBM1

**Figure 3:**
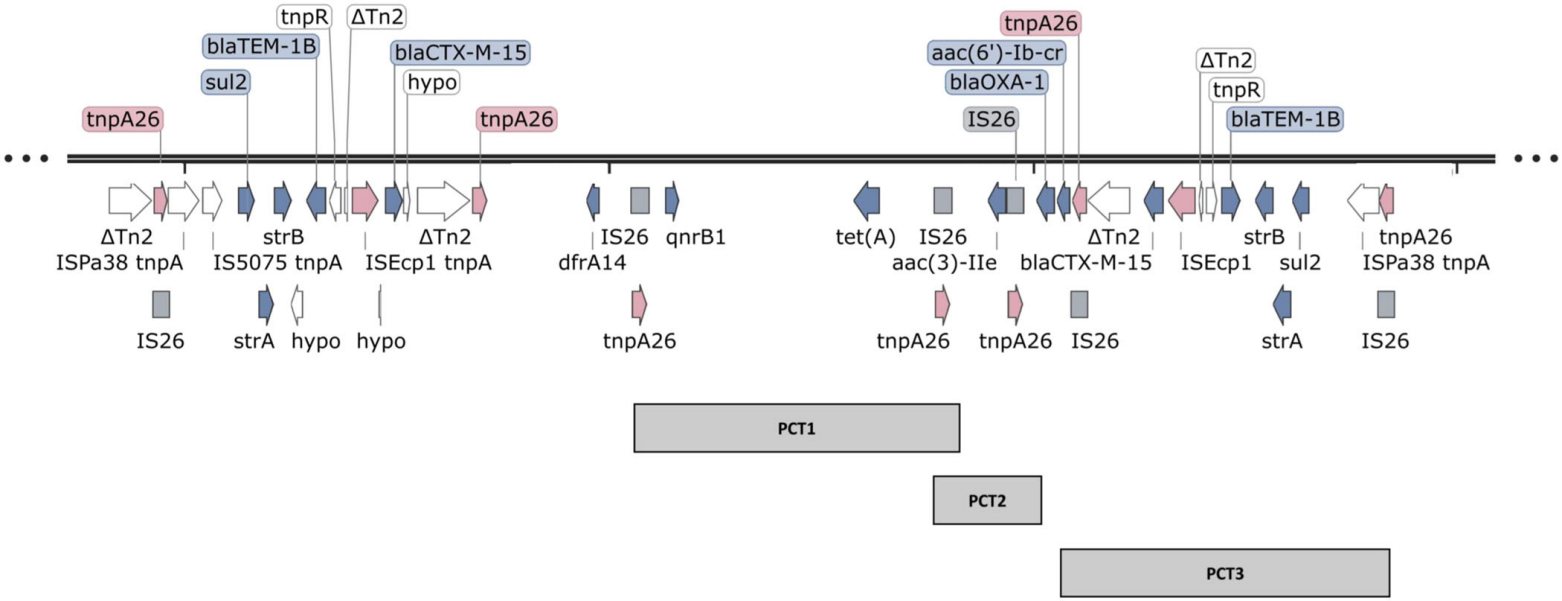
Resistance genes and their association with IS*26*. Two resistance regions encoding 5 antimicrobial resistance (AMR) genes: *sul2, strA, strB, bla*_TEM‐1B_, and *bla*_CTX‐M‐15_, respectively are present on pKBM1. The first resistance region, 15,865‐bp, lacks an IRL from the second IS*26*. This resistance region is therefore not a complete pseudo‐compound transposon (PCT). The second 15,301‐bp region (PCT3) encoding the same five AMR genes has two complete directly orientated IS*26* with complete IRL and IRR sequences. IS*26* is shaded as a grey rectangle, and its corresponding transposase gene, *tnpA26* is shaded pink. Resistance genes are shown in blue, while other genes present in the resistance region are shown in white. Gene arrows correspond to the direction of transcription. The resistance region was annotated using Prokka v1.14.6 (Seemann, 2014) and visualized using SnapGene v.7.1 (https://www.snapgene.com/).

Three complete IS*26* PCTs encoding AMR genes are present in pKBM1 (Table 2 and Figure 3). An IS*26* is shared between PCT1 and PCT2, forming a tandem array of IS*26* PCTs. The absence of an IRL for the right IS*26* downstream of *sul2, strA, strB, bla*_TEM‐1B_, and *bla*_CTX‐M‐15_ disrupts the formation of a series of 4 consecutive IS*26* PCTs (Figure 3).

**Table 2:** Intact IS*26* pseudo‐compound transposons (PCTs) encoding antimicrobial resistance (AMR) genes identified within pKBM1.

Intact IS*26* PCT	Position in contig pKBM1	Length (bp)	AMR genes
PCT1	70034‐85148	15,115	*qnrB1, tet(A)*
PCT2	66614‐70852	4239	*aac(3)‐IIe*
PCT3	49127‐64427	15,301	*sul2, strA, strB, bla*_TEM‐1B_, *bla*_CTX‐M‐15_

The AMR genes: *sul2, strA, strB, bla*_TEM‐1B_, and *bla*_CTX‐M‐15_ are found in two unique locations on pKBM1. The first set of these five AMR genes are found within a 15,865‐bp structure. The resistance block has an IS*26* IRL at the outermost left end, and an IS*26* IRR at the outermost right end, bracketing the set of resistance genes. While the outermost IS*26* IRL and IRR sequences are present, the IRR of Tn2 (accession: KT00254), located downstream of IS*Ecp*1‐*bla*_CTX‐M‐15_, interrupts the IRL of the second directly orientated IS*26*, removing the first 27 nucleotides. The 15,865‐bp structure therefore lacks two complete IS*26*s enclosing the set of five resistance genes. As such, the structure does not resemble a complete IS*26* PCT.

### 3.5 pEBM1 IS*26* PCT structure

**Figure 4:**
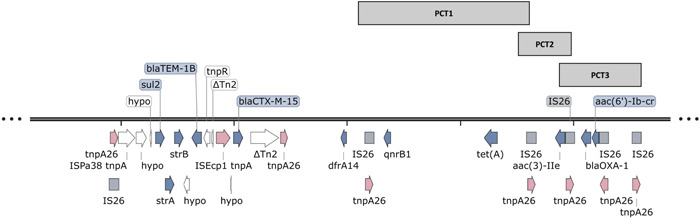
Resistance genes and their association with IS*26* pseudo‐compound transposons (PCTs) from pEBM1. Three complete IS*26* PCTs are present in pEBM1. These are indicated as solid grey rectangles with their PCT number. IS*26* is shared between two of the three IS*26* PCTs, forming a tandem IS*26* PCT array. Five resistance genes: *sul2, strA, strB, bla*_TEM‐1B_, and *bla*_CTX‐M‐15_, respectively were found within a 15,683 bp structure from pEBM1. IS*26* is shaded as a grey rectangle, and its corresponding transposase gene, *tnpA26* is shaded pink. Resistance genes are shown in blue, while other genes present in the resistance region are shown in white. Gene arrows correspond to the direction of transcription. pEBM1 accession: CP120235.2. The resistance region was annotated using Prokka v1.14.6 (Seemann, 2014) and visualized using SnapGene v.7.1 (https://www.snapgene.com/).

A complex mosaic of IS*26* sequences is found bracketing the resistance genes in pEBM1. Three intact IS*26* PCTs encoding resistance genes are present. For each IS*26* copy at the boundary, complete IRL and IRL sequences are found. Two IS*26*s are shared between the three PCTs (Table 3 and Figure 4).

**Table 3:** Intact IS*26* pseudo‐compound transposons (PCTs) encoding antimicrobial resistance (AMR) genes identified within pEBM1.

Intact IS*26* PCT	Position in contig pKBM1	Length (bp)	AMR genes
PCT1	168,761–183,878	15,118	*qnrB1, tet(A)*
PCT2	183,059–187,298	4240	*aac(3)‐IIe*
PCT3	186,479–193,185	6707	*bla*_OXA‐1_, *aac(6’)‐Ib‐cr*

**Figure 5:**
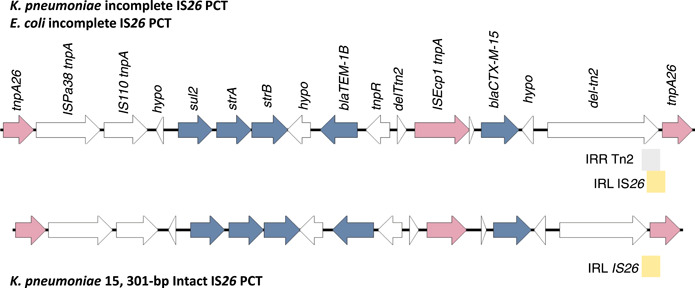
Comparison between incomplete IS*26 bla*_CTX‐M‐15_ pseudo‐compound transposons (PCTs) from both *Klebsiella pneumoniae* and *Escherichia coli*. The gene organization is similar between the IS*26* PCT in *K. pneumoniae* and the incomplete IS*26* PCT structures in *E. coli* and *K. pneumoniae*. Notably, for the incomplete structures (top), the IRR of Tn2 (accession: KT002541) is present and interrupts IS*26*, removing the first 27 nucleotides, including the 14 bp IRL of IS*26*. The interruption is depicted as a grey rectangle, truncating the IRL of IS*26* (yellow rectangle). In comparison, a complete IS*26* is found for the intact IS*26* PCT structure (bottom). The incomplete PCTs contain a 588 bp insertion, whereby the longer Tn2 fragment carries a disrupted Tn2 transposase of 849 amino acids versus the 670 amino acids present in the complete 15,301 bp IS*26* PCT. Genome comparisons were performed using Easyfig v.2.2.5 (Sullivan et al., 2011).

Similar to the resistance region in pKBM1, five AMR genes including *sul2, strA, strB, bla*_TEM‐1B_, and *bla*_CTX‐M‐15_, respectively were found within a 15,683‐bp resistance region bracketed by the IS*26* transposase genes, *tnpA26* on pEBM1. The IRR of Tn2 (KT002541) downstream of IS*Ecp*1‐*bla*_CTX‐M‐15_ interrupts the IS*26* sequence, truncating the second directly orientated IS*26* sequence by 27 nucleotides, removing the 14‐bp IRL. Both pEBM1 and pKBM1 share the same disruption in IS26 (Figure 5). The same genomic signature from both IS*26*‐PCT regions may infer their derivation from the same plasmid. The truncation of the IS*26* sequence disrupts the tandem IS*26* PCT structure. For pEBM1, in addition to the disruption of the complete IS*26* PCT encoding *sul2, strA, strB, bla*_TEM‐1B_, and *bla*_CTX‐M‐15_, the absence of the IRL for the second directly orientated IS*26* prevents an intact IS*26* PCT enclosing the *dfrA14* gene. This disruption prevents five intact IS*26* PCTs from being organized in tandem structure.

### 3.6 Resistance region comparison against pKPN3‐307_typeA

**Figure 6:**
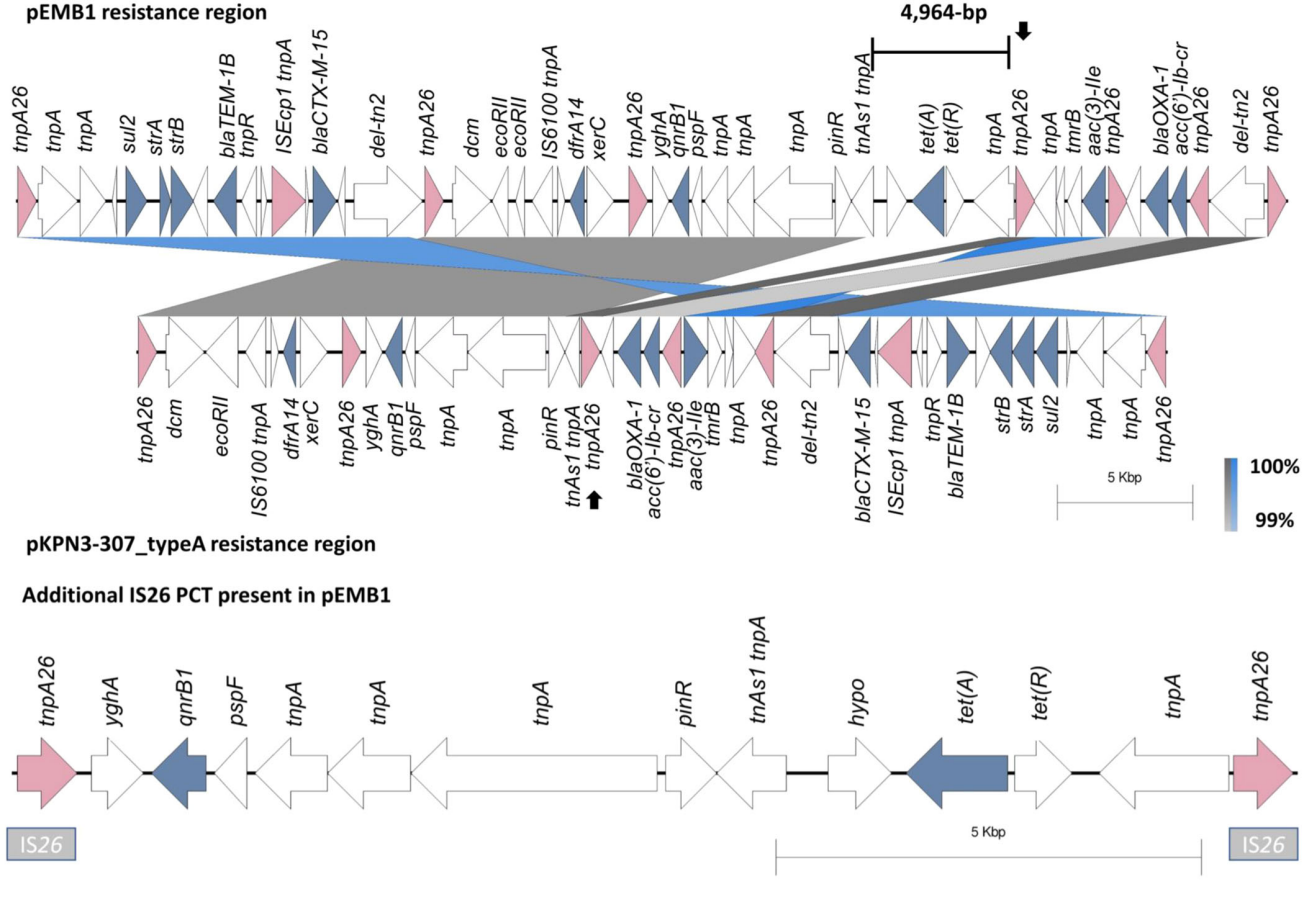
Comparison between the 47,012‐bp resistance region in pEBM1 and the 38,107‐bp resistance region in pKPN3‐307_typeA. A 14,486‐bp resistance region (first blue section from the left side) harboring the five AMR genes: *sul2, strA, strB, bla*_TEM‐1B_, and *bla*_CTX‐M‐15_ is shared between the two plasmids. This region is inverted relative to the resistance region in pEBM1. In plasmid, pKPN3‐307_typeA, a 15,311‐bp IS*26* pseudo‐compound transposons (PCT) with complete IRL and IRR for each IS*26* copy (accession: X00011) is present, while pEBM1 lacks an IRL for the second copy of the directly oriented IS*26* due to a 27‐nucleotide truncation via the IRR of Tn2 (accession: KT002541). The two plasmids share a 16,348‐bp region encoding both the AMR genes *dfrA14* and *qnrB1*, respectively. Upstream of the shared region, pEBM1 contains a 4964‐bp insertion including the *tet(A)* gene. This gene is placed between a shared IS*26* in the 16‐kb block, and a shared IS*26* upstream of the 4964‐bp insertion, indicated by an arrow. This organization forms a complete 15,118‐bp IS*26* PCT encoding both *qnrB1* and *tet(A)*, respectively (bottom). Relative to pKPN3‐307_typeA, a relocation of *bla*_OXA‐1_, *aac(6’)‐Ib‐cr* with inwardly facing copies of IS*26* encoding *tnpA26*, and an inversion of the IS*26* PCT encoding *aac(3)‐IIe* is present in pEBM1. Inversions are depicted in blue, and genes with the same orientation are depicted in grey. Genome comparisons were performed using Easyfig v.2.2.5 (Sullivan et al., 2011).

The resistance region from pEBM1 shares similarity against the internationally prevalent plasmid FIB(K) pKPN3‐307_typeA (accession: KY271404.1) commonly identified in *K. pneumoniae* ST307 (Hawkey et al., 2022). pEBM1 harbors a 47,012‐bp resistance region encoding 11 AMR genes between abutting IS*26* copies, while pKPN3‐307_typeA has a nucleotide stretch of 38,107‐bp encoding 10 AMR genes between outer IS*26* copies. The two plasmids share the same 10 AMR genes: *sul2, strA, strB, bla*_TEM‐1B_, *bla*_CTX‐M‐15_, *dfrA14, qnrB1, aac(3)‐IIe, bla*_OXA‐1_, and *aac(6’)‐Ib‐cr*, respectively. pEBM1 additionally carries the *tet(A)* resistance gene. The resistance region of pKPN3‐307_typeA shares 100% query coverage and 99.95% identity against the region harboring resistance genes in pEBM1. Relative to the resistance encoding loci in pEBM1, the resistance section from pKPN3‐307_typeA has 2 inversions including the region encoding *bla*_CTX‐M‐15_, and multiple relocations (Figure 6). Notably, beyond a shared 16,348‐bp region encoding the AMR genes *dfrA14* and *qnrB1*, an insertion of 4964‐bp including the *tet(A)* is present between a shared IS*26* in pEBM1 forming a separate 15,118‐bp IS*26* PCT encoding *qnrB1‐tet(A)* in pEBM1. The same IS*26* PCT encoding *qnrB1*‐*tet(A)* is found in pKBM1.

## 4 DISCUSSION

Two plasmids with the same genotypic resistance profile were obtained from the same CSU sample. The plasmids appear related suggesting HGT has occurred during infection, leading to a polymicrobial infection. The unique signature where the Tn2 fragment interrupts the first 27 nucleotides of the second directly oriented IS*26* copy in the PCT encoding *bla*_CTX‐M‐15_ is present in both plasmids. The plasmids also share the same AMR genes with the same organization in the resistance loci, except for an additional IS*26*‐harboring *bla*_CTX‐M‐15_ PCT in pKBM1. This may infer that the plasmids are related and derived from a common source. A common origin is also supported by an identical shared FIB(K) replicon, the presence of the *tra* operon in both plasmids, high query coverage of pKBM1 against pEBM1, and their isolation at the same time from the same source. Together, this suggests HGT may have occurred before subsequent plasmid re‐arrangements.

Plasmids that comprise the urinary microbiota, known as the urobiome, remain poorly characterized. Our results using long‐read sequencing technology, further expand the known plasmid content from multiple bacterial strains occupying the urobiome. In line with a previous report that investigated plasmid content from urinary isolates, similar or identical plasmids were found to be shared between urobiome isolates from different genera, including a 1546‐bp plasmid sequence found in two *E. coli* and one *K. pneumoniae* strain (Johnson et al., 2022). Shared plasmid sequences were also found between *E. coli* and *Nosocomiicoccus ampullae, Staphylococcus aureus* and *Staphylococcus epidermidis*, and *Alloscardovia omnicolens* and *Lactobacillus gasseri* (Johnson et al., 2022). This suggests plasmid exchange can occur between different species that comprise the urobiome.

Notably, both plasmids carried copper resistance genes: *copA, copB, copC*, and *copD*, and the copper cation efflux system *cusSRCFBA*. Recently, a *K. pneumoniae* strain, KpnU95, harboring the same copper resistance genes on pKpnU95 had a faster growth rate in the presence of copper ions relative to the same strain cured of pKpnU95 (Gancz et al., 2021). Copper‐resistance systems in bacterial strains are advantageous for uropathogenic *K. pneumoniae* strains protecting high urinary copper concentrations (Hyre et al., 2017). Conservation of this region in both plasmids may be important for persistent UTIs.

Both plasmids share similarity with the AMR encoding region of the internationally prevalent plasmid, pKPN3‐307_typeA‐like (accession: KY271404.1) (Wyres et al., 2019). pEBM1 and pKBM1 additionally carry the *tet(A)* gene within the shared AMR region. The *tet(A)* gene is bordered by 2 IS*26* copies with perfect 14‐bp IRL/IRRs, forming a complete IS*26* PCT, that is not present in the resistance loci from pKPN3‐307_typeA. This region may have been formed via IS*26*‐mediated deletions/insertions within the respective AMR region. This PCT could relocate both the *qnrB1* and the *tet(A)* genes via either the copy‐in route to a separate replicon in the same cell, as described in Harmer et al., 2020, or via the targeted conservative cointegration method (Harmer & Hall, 2017; Harmer et al., 2014).

Two copies of the five AMR genes: *sul2, strA, strB, bla*_TEM‐1B_, and *bla*_CTX‐M‐15_ encoding resistance to clinically important antibiotics were detected in pKBM1. While both the copy‐in and targeted conservative cointegration routes are mechanisms that can disseminate AMR genes bounded between IS*26* copies, the duplication of these genes and the formation of a novel PCT may have occurred via large‐scale plasmid rearrangement. Immediately upstream of a shared IS*26* between pKBM1 and pEBM1, a 52.8‐kb fragment from pEBM1 is located. This integration has formed a novel IS*26*‐PCT with perfect 14‐bp TIRs at each end of the IS*26*. This IS*26*‐PCT may be able to undergo subsequent relocation to target DNA molecules via both the copy‐in and targeted conservative co‐integration routes. Indeed, a 15,309‐bp IS*26* PCT encoding the same five AMR genes with the same organization has been found in pAMA1416 (accession: MG462728.1) from *E. coli* strain AMA1416. This PCT was found in a unique genomic context relative to the 15,301‐bp IS*26* PCT in pKBM1, suggesting the IS*26* PCT is mobile.

An array of resistance genes with abutting IS*26* copies are present in both pKBM1 and pEBM1. This organization likely reflects how the complex resistance loci (CRL) was formed. Translocatable units (TUs) may have preferentially targeted existing IS*26* copies in a conservative manner forming a multimeric array of IS*26* and resistance genes as described in Harmer et al. (2014). Separate TU‐encoding AMR genes may be able to form this region and integrate into target replicons including chromosomes and plasmids. Indeed, separate TUs were identified from 19,774‐bp CRL encoding AMR genes bracketed between IS*26* copies (Chowdhury et al., 2019).

The *oriT* site is present in pEBM1 alongside a complete *tra* operon. This may suggest onward dissemination of pEBM1 is possible to receptive strains. *bla*_CTX‐M‐15_ encoding strains contribute to the epidemic success of pathogens in clinical environments (Hansen et al., 2020; Hawkey et al., 2022). Their presence on a plasmid with conjugative transfer machinery may provide a mechanism for the onward dissemination of broad‐spectrum resistance from EC_01/KC1‐like strains.

While we conducted a nanopore‐only approach for genome assembly, we recognize the consensus accuracy may be lifted using both short‐ and long‐reads for hybrid genome assembly. Tools such as PlasmidSPAdes (https://github.com/ablab/spades#plasmid) enable plasmid assembly from whole genome sequencing (WGS) data sets including long‐ and short‐reads. However, the high coverage achieved for each plasmid, the high threshold for resistance gene identification on circularized plasmids, and the corresponding genotype‐to‐phenotype relationship support the use of WGS using nanopore reads only. Crucially, nanopore assembly allowed the genomics architecture of resistance genes and their associated insertion sequences to be resolved.

## 5 CONCLUSION

We identified two bacterial strains with the same phenotypic resistance profile. The similarity between both plasmids isolated from each strain suggests a common derivation followed by subsequent plasmid rearrangement, including cointegrate formation with a pSCU‐485‐1‐like plasmid in pEBM1, and the formation of an additional IS*26* PCT encoding *bla*_CTX‐M‐15_ in pKBM1. Catheter biofilm may have provided an environment whereby EC_01/KC1 strains had close cell–cell contact, leading to conjugation. HGT of plasmids and subsequent plasmid rearrangement is likely to have occurred during infection. Clinical interventions to prevent healthcare‐acquired bacteriuria include limiting indwelling catheter use or seeking alternatives such as external collection devices. Other approaches include modifying catheters using antifouling agents such as polyethylene glycol or hydrogels, which restrict bacterial colonization, thereby preventing biofilm formation (Andersen & Flores‐Mireles, 2019). In cases where catheters are required, their use should be discontinued as soon as is clinically practicable to help reduce the spread of antibiotic‐resistant strains. Our findings further highlight the importance of careful antimicrobial stewardship to limit antibiotic exposure in catheterized patients, and to help reduce selection pressure for the emergence and spread of resistance.

## AUTHOR CONTRIBUTIONS

**Stephen Mark Edward Fordham**: Conceptualization (equal); data curation (equal); formal analysis (equal); investigation (equal); methodology (equal); validation (equal); writing—original draft (equal); writing—review and editing (equal). **Magdalena Barrow**: Investigation (equal); methodology (equal); resources (equal). **Anna Mantzouratou**: Funding acquisition (equal); project administration (equal); supervision (equal); writing—review and editing (equal). **Elizabeth Sheridan**: Data curation (equal); formal analysis (equal); funding acquisition (equal); project administration (equal); supervision (equal); writing—review and editing (equal).

## CONFLICT OF INTEREST STATEMENT

None declared.

## ETHICS STATEMENT

Ethics approval for project ID 27771 was granted by the Bournemouth University Ethics Committee.

## Data Availability

The data that support the findings of this study are openly available on Genbank. The *K. pneumoniae* chromosome (KC1) and plasmid (pKBM1) assembly can be found on GenBank under the accession numbers: CP119564.1, and CP119565.2. The *E. coli* chromosome (EC_01) and plasmid (pEBM1) assembly can be found on GenBank under the accession numbers: CP120234.1, and CP120235.2. All data generated or analyzed during this study are included in this published article. The *K. pneumoniae* chromosome (KC1) and plasmid (pKBM1) assembly can be found on GenBank under the accession numbers: CP119564.1, and CP119565.2; BioProject PRJNA942233: https://www.ncbi.nlm.nih.gov/bioproject/PRJNA942233. The *E. coli* chromosome (EC_01) and plasmid (pEBM1) assembly can be found on GenBank under the accession numbers: CP120234.1, and CP120235.2; BioProject PRJNA942235: https://www.ncbi.nlm.nih.gov/bioproject/PRJNA942235.
